# Globally altered microstructural properties and network topology in Rasmussen’s encephalitis

**DOI:** 10.1093/braincomms/fcad290

**Published:** 2023-11-01

**Authors:** Nina R Held, Tobias Bauer, Johannes T Reiter, Christian Hoppe, Vera C W Keil, Alexander Radbruch, Christoph Helmstaedter, Rainer Surges, Theodor Rüber

**Affiliations:** Department of Epileptology, University Hospital Bonn, 53127 Bonn, Germany; Department of Epileptology, University Hospital Bonn, 53127 Bonn, Germany; Department of Epileptology, University Hospital Bonn, 53127 Bonn, Germany; Department of Epileptology, University Hospital Bonn, 53127 Bonn, Germany; Department of Neuroradiology, University Hospital Bonn, 53127 Bonn, Germany; Department of Radiology and Nuclear Medicine, Amsterdam UMC Location Vrije Universiteit Amsterdam, 1081 HV Amsterdam, Netherlands; Amsterdam Neuroscience, Brain Imaging, Amsterdam UMC, 1081 HV Amsterdam, Netherlands; Cancer Center Amsterdam, Brain Tumor Center Amsterdam, Amsterdam UMC, 1081 HV Amsterdam, Netherlands; Department of Neuroradiology, University Hospital Bonn, 53127 Bonn, Germany; Department of Epileptology, University Hospital Bonn, 53127 Bonn, Germany; Department of Epileptology, University Hospital Bonn, 53127 Bonn, Germany; Department of Epileptology, University Hospital Bonn, 53127 Bonn, Germany

**Keywords:** connectomes, diffusion-tensor imaging, fixel-based analysis, inflammation, paediatric epilepsy

## Abstract

Rasmussen’s encephalitis is an immune-mediated brain disorder characterised by progressive unilateral cerebral atrophy, neuroinflammation, drug-resistant seizures and cognitive decline. However, volumetric changes and epileptiform EEG activity were also observed in the contralateral hemisphere, raising questions about the aetiology of contralateral involvement. In this study, we aim to investigate alterations of white matter integrity, structural network topology and network efficiency in Rasmussen’s encephalitis using diffusion-tensor imaging. Fourteen individuals with Rasmussen’s encephalitis (11 female, median onset 6 years, range 4–22, median disease duration at MRI 5 years, range 0–42) and 20 healthy control subjects were included. All subjects underwent T1-weighted structural and diffusion-tensor imaging. Diffusion-tensor images were analysed using the fixel-based analysis framework included in the MRtrix3 toolbox. Fibre density and cross-section served as a quantitative measure for microstructural white matter integrity. T1-weighted structural images were processed using FreeSurfer, subcortical segmentations and cortical parcellations using the Desikan-Killiany atlas served as nodes in a structural network model, edge weights were determined based on streamline count between pairs of nodes and compared using network-based statistics. Global efficiency was used to quantify network integration on an intrahemispheric level. All metrics were compared cross-sectionally between individuals with Rasmussen’s encephalitis and healthy control subjects using sex and age as regressors and within the Rasmussen’s encephalitis group using linear regression including age at onset and disease duration as independent variables. Relative to healthy control subjects, individuals with Rasmussen’s encephalitis showed significantly (family-wise-error-corrected *P* < 0.05) lower fibre density and cross-section as well as edge weights in intrahemispheric connections within the ipsilesional hemisphere and in interhemispheric connections. Lower edge weights were noted in the contralesional hemisphere and in interhemispheric connections, with the latter being mainly affected within the first 2 years after disease onset. With longer disease duration, fibre density and cross-section significantly (uncorrected *P* < 0.01) decreased in both hemispheres. In the contralesional corticospinal tract, fibre density and cross-section significantly (uncorrected *P* < 0.01) increased with disease duration. Intrahemispheric edge weights (uncorrected *P* < 0.01) and global efficiency significantly increased with disease duration in both hemispheres (ipsilesional *r* = 0.74, *P* = 0.001; contralesional *r* = 0.67, *P* = 0.012). Early disease onset was significantly (uncorrected *P* < 0.01) negatively correlated with lower fibre density and cross-section bilaterally. Our results show that the disease process of Rasmussen’s encephalitis is not limited to the cortex of the lesioned hemisphere but should be regarded as a network disease affecting white matter across the entire brain and causing degenerative as well as compensatory changes on a network level.

## Teaser

Held, Bauer *et al*. evaluated white matter microstructural topology and network connectivity in Rasmussen's encephalitis. Their results demonstrate a prominent involvement of interhemispheric fibres in disease progression and remodelling at both microstructural and network levels, leading to the conclusion that Rasmussen's encephalitis should be considered a whole-brain network disorder.

## Graphical Abstract

**Graphical Abstract:**
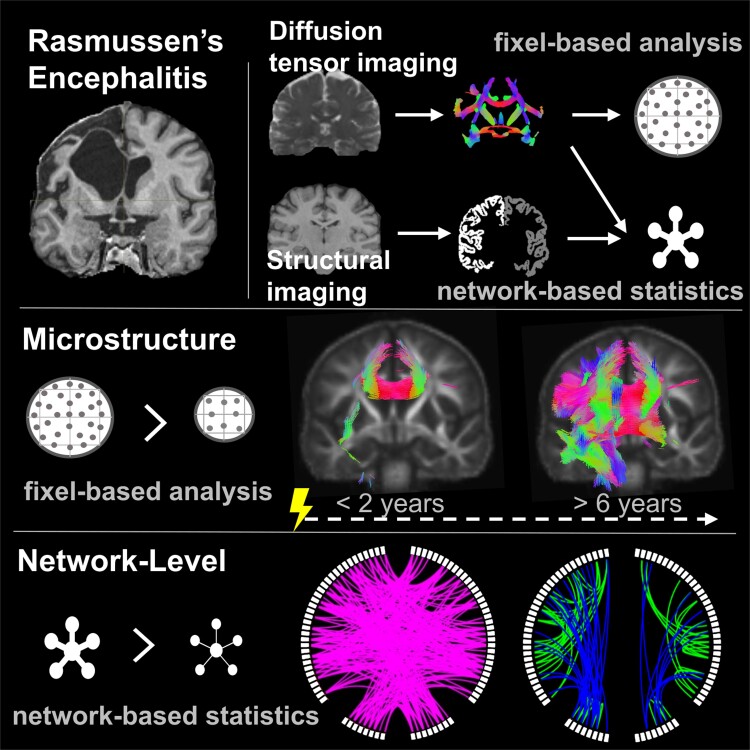

## Introduction

Rasmussen’s encephalitis (RE) is a rare immune-mediated brain disorder mainly affecting children. It is characterised by progressive unilateral cerebral atrophy and neuroinflammation, drug-resistant seizures and cognitive decline.^1^ The presumably unihemispheric manifestation is its most characteristic feature and is under scrutiny. Previous research has revealed that also the contralesional, often denoted as the ‘unaffected’ hemisphere, is subject to volumetric changes. However, these results are ambiguous, and both an increase^2^ and a decrease^3^ in contralesional volume have been shown. On electrophysiological grounds, there is also growing evidence that epileptiform activity is not restricted to the ipsilesional hemisphere, but can also be observed in the contralesional hemisphere.^4-7^ Several hypotheses to explain contralesional involvement under the umbrella of secondary degeneration have been discussed. These include Wallerian degeneration, which is a concept of degeneration in distal parts of damaged axons,^8^ and transhemispheric diaschisis, which defines degeneration in brain areas contralateral to the primary disease focus as a consequence of reduced afferent input from these lesioned brain regions.^9-11^

Both scenarios imply involvement of the connecting white matter tracts, which, so far, have not yet been investigated in RE. In this study, we aimed to investigate connectivity alterations specifically in RE based on diffusion-tensor imaging (DTI) using a 3-fold approach: First, we sought to assess white matter microstructural integrity by applying fixel-based analysis (FBA), which is an advanced framework for DTI analysis that is highly sensitive to fibre-specific microstructural properties and independent of local fibre geometry. Fixels are distinct fibre populations within a voxel, and thus, in contrast to most conventional DTI-based analyses, FBA provides fibre-specific quantitative measures for microstructural integrity, while being unaffected by signal noise due to crossing fibres or other complex fibre configurations possibly contained in one voxel.^12,13^ Second, we aimed to investigate whether possible alterations on a tract-level translate into altered connectivity on a network level, which can be measured based on structural connectomes derived from tractography.^14^ Third, we sought to evaluate network efficiency to explore the impact of possible local network-level alterations on the efficiency of the overall hemispheric network.

We hypothesised that this 3-fold strategy would reveal microstructural correlates of the natural disease course of RE in the ipsilesional hemisphere, microstructural and network-level alterations in transhemispheric connections that would explain contralesional involvement and compensatory network-level restructuring.

## Materials and methods

### Study group

We retrospectively ascertained imaging and clinical data from all individuals with RE diagnosed according to the diagnostic criteria of the 2005 European consensus statement^1^ who were treated at the Department of Epileptology of the University Hospital Bonn between 2000 and 2013 and had at least one DTI and T1-weighted MRI scan available. From 2014 until 2022, all individuals with RE^1^ who were treated at the Department of Epileptology of the University Hospital Bonn and did not have any contraindication for MRI were prospectively enrolled. Further clinical and demographic characteristics are provided in subsection *RE group* and Table 1. As a control group, DTI and T1-weighted MRI scans from 20 individuals with no history of neurological or psychiatric diseases were selected from an in-house database. The study was approved by the Institutional Review Board of the University Hospital Bonn. We obtained written informed consent from all participants or their legal guardians according to the Declaration of Helsinki.

**Table 1:** Demographic data

	RE	Healthy controls	*P*
Subjects, *n*	14	20	n/a
Sex female, *n* (%)	11 (79)	13 (65)	0.64
Age at MRI, years; median (range)	17.5 (7–49)	23.5 (9–49)	0.74
Age at onset, years; median (range)	6 (4–22)	n/a	n/a
Disease duration at MRI, years; median (range)	8.5 (0–42)	n/a	n/a
Number of ASM; median (range)	6 (0–10)	n/a	n/a
Left hemisphere affected, *n* (%)	8 (57)	n/a	n/a

*P*-values refer to unpaired, two-tailed *t*-test or chi-squared-test as appropriate. ASM, anti-seizure medication.

### MRI acquisition and pre-processing

DTI and T1-weighted MRI were acquired at the Life & Brain Center, University Hospital Bonn, using a 3 T MRI scanner (Magnetom Trio, Siemens Healthineers, Erlangen, Germany, years 2014–22) and at the Department of Neuroradiology, University Hospital Bonn, using a 3 T MRI scanner (Philips Healthcare Ingenia 3.0 T, Best, Netherlands, years 2011–13). Acquisition parameters differ between both scanning sites and are detailed in Supplementary Table 2. DTI image pre-processing was performed using the FMRIB Software Library 6.0^15^ (FSL) and the MRtrix3 package.^16^ Pre-processing included denoising, unringing, correcting for susceptibility-induced geometric distortions by generating a synthesised *b*0 and applying the FSL tools *topup* and *eddy.*^17^

### Fixel-based analysis

FBA is a framework for DTI analysis that estimates microstructural properties of individual fibre populations within a voxel, denoted as a fixel. For FBA, all MRI images of individuals with RE and right-sided ipsilesional hemispheres and the same proportion of control subjects were flipped along the x-axis. FBA was performed according to the recommended methodology.^13^ All pre-processed DTI scans were upsampled to an isotropic voxel size of 1.25 mm^3^ and an average single-shell response function was calculated across all people with RE and control subjects. Fibre orientation distributions (FODs) were then estimated using constrained spherical deconvolution and the multi-shell–multi-tissue algorithm to take advantage of the hard non-negativity constraint even with single-shell data. We then generated a study-specific FOD population template using all individual FOD images as input and registered all subjects into the template space using a non-linear warp. A template mask was computed based on the intersection of all individual masks in template space to ensure that only fixels present in all subjects were analysed. After performing global intensity normalisation and estimating a group average spherical deconvolution response function, FOD amplitudes can be used to quantify the intra-axonal volume of axons aligned in that direction. This measure is also referred to as fibre density (FD).^18^ Based on the non-linear warps generated during the registration of subjects to the template space we calculated the fibre bundle cross-section (FC) of all fixels.^13^ We computed fibre density and cross-section (FDC) as the combined metric by multiplication of FD and FC to be used as a quantitative measure for microstructural integrity. To perform group statistical analysis of FC, we calculated the log(FC) to ensure data are distributed normally and centred around zero. Whole-brain probabilistic tractography was then carried out on the population template, selecting streamlines of a length between 10 and 250 mm and a maximum angle of 22.5°. Subsequently, we performed spherical deconvolution informed filtering of tractograms (SIFT) to reduce bias in tractogram densities which decreased the number of streamlines from an initial 20 million to 2 million using the *tcksift* algorithm.^19^

### Connectome construction

The FreeSurfer (v6.0) *recon-all* algorithm was used to segment cortical and subcortical structures based on the Desikan-Killiany atlas.^20,21^ The T1-weighted images were then affinely registered to the individual b0 diffusion image, and the parcellation images were transformed to the diffusion space using FSL FLIRT.^22^ The pre-processed DTI images were segmented using the five-tissue-segmentation algorithm *5ttgen* provided by Mrtrix3. Note that in contrast to the FBA, we estimated a unique response function for each participant and FODs were calculated using spherical deconvolution and the multi-shell–multi-tissue algorithm in the individual subject space. Anatomically constrained tractography was performed on the FOD images, and we applied SIFT to reduce the number of streamlines from 10 million to 5 million.^23^ Weighted, undirected connectomes were constructed using the *tck2connectome* command^24^ while scaling each contribution to the connectome edge by the inverse of its two node volumes^14^ to decrease bias caused by a higher probability for a larger parcel to be intersected by any streamline. This method offers the advantage of covering anatomically compact or atrophic areas that inherently have a limited grey matter–white matter interface for streamline initiation. We used the Brain Connectivity Toolbox to calculate the global efficiency of each hemisphere separately for all participants.^25^

### Statistical analysis

**Figure 1:**
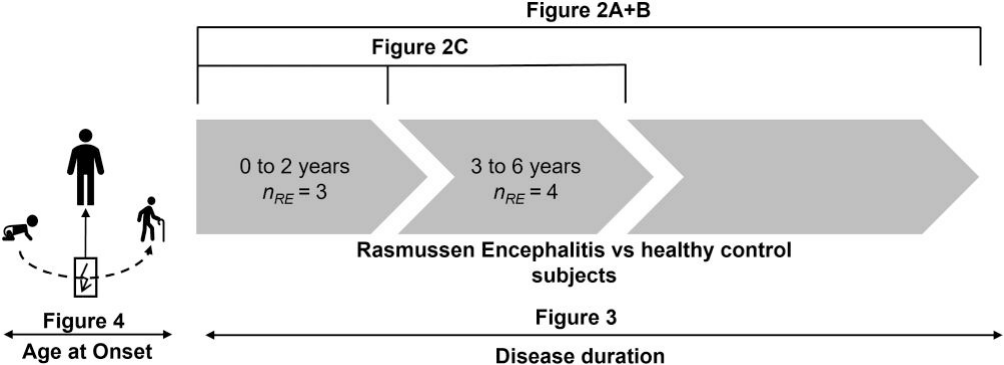
**Statistical comparisons.** Figure 2A and B compare all individuals with Rasmussen’s encephalitis and all healthy control subjects. Figure 2C shows the comparison between sub-groups of individuals with RE between 0 to 2 years, and 3 to 6 years after the onset of the disease and control subjects. Figure 3 shows a linear regression within the RE group as a function of disease duration, while Fig. 4 shows a linear regression as a function of age at disease onset.

Statistical analysis on FD, log(FC), FDC and edge weights (EWs) was performed using two general linear models. First, individuals with RE and 20 healthy control subjects were compared regarding group differences in FD, log(FC), FDC or EWs, including sex and age at MRI as independent variables, while demeaning age as a continuous variable in order to improve matrix conditioning. Second, for the RE group, we used a model with either FD, log(FC), FDC or EWs as dependent variables, and disease duration and age at onset as independent variables. Parameter estimates were tested for significance using two-tailed *t*-tests. Continuous independent variables were demeaned to improve conditioning. See Fig. 1 for a schematic of the linear models applied in this study.

**Figure 2:**
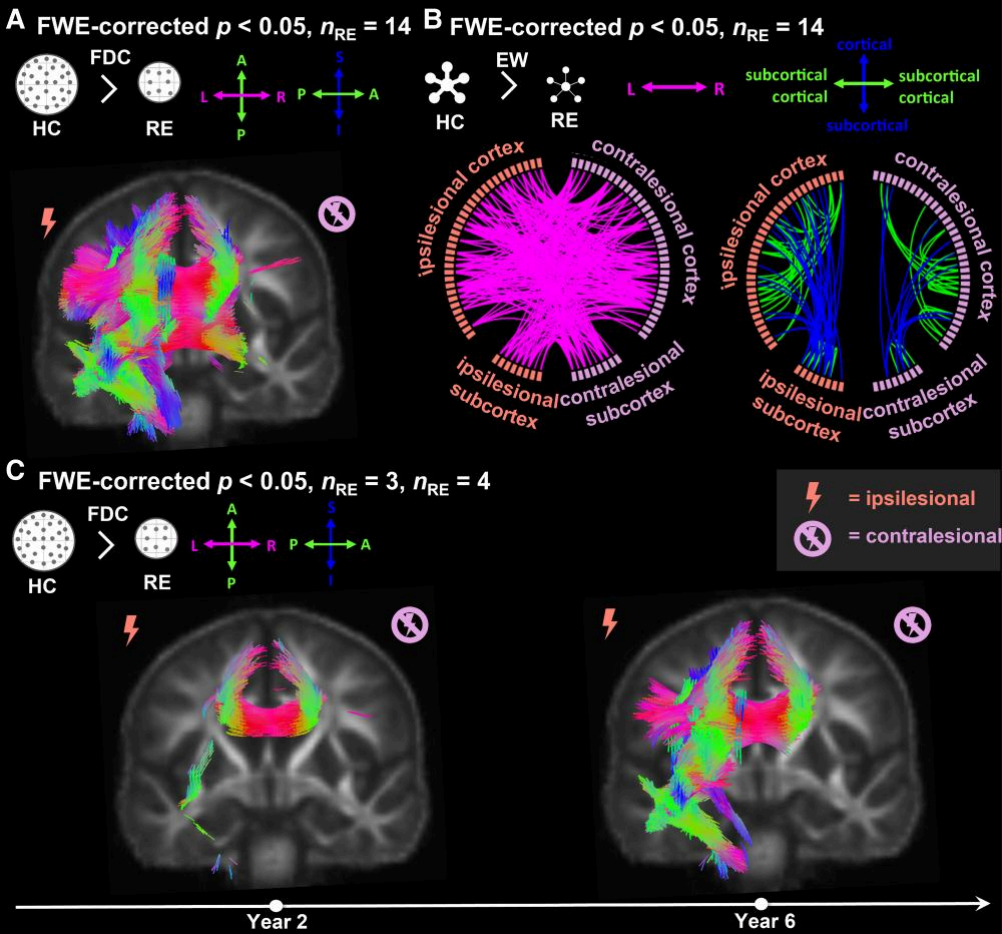
**Group-wise comparison between individuals with Rasmussen’s encephalitis (RE) and 20 healthy control subjects.** (**A**) Fixels with significantly [family-wise error (FWE) corrected *P* < 0.05] lower fibre density and cross-section (FDC) in individuals with RE compared to healthy control subjects (*n*_RE_ = 14). (**B**) Significantly lower (FWE-corrected *P* < 0.05) EW in individuals with RE compared to healthy control subjects (*n*_RE_ = 14). (**C**) Fixels with significantly lower FDC relative to control subjects 2 years (*n*_RE_ = 3), and between 2 and 6 years (*n*_RE_ = 4) after disease onset. In (**A/C**), significant fixels are coloured by direction and shown on the population-specific fractional anisotropy template sliced in the coronal plane. RE, Rasmussen’s encephalitis; HC, healthy control subjects; FDC, fibre density and cross-section; EW, edge weight; FWE, family-wise error; *n*_RE_, number of individuals with Rasmussen’s encephalitis.

For testing of fixel-based metrics, we used connectivity-based fixel enhancement and non-parametric permutation testing over 5000 permutations if not indicated otherwise to compute family-wise error (FWE)-corrected *P*-values for each fixel.^13^

For network-based statistics on structural connectomes, we performed non-parametric permutation testing over 5000 permutations using the threshold-free network-based statistics algorithm^26^ if not indicated otherwise to compute FWE-corrected *P*-values for each connectome edge. Anatomical labels for connectome edges are provided in Supplementary Fig. 1. We performed Welch's two-sided unequal variance *t*-test to test for group differences regarding global efficiency between control, ipsilesional and contralesional hemispheres. As there were no significant differences between the right and left hemispheres in the control group, left and right hemispheres of the control group were pooled. Pearson's correlation coefficient was calculated for the relationship between global efficiency and disease duration using the *SciPy* module in *Python*. We regard FWE-corrected *P* < 0.05 as statistically significant. Due to the small sample size (*n* = 14) for within-group comparisons, in this scenario, uncorrected *P* < 0.01 was used to infer statistical significance.

## Results

### RE cohort

Between 2000 and 2022, in total 68 individuals with RE were treated at our department. In five cases, DTI scans were performed for surgery planning at the Department of Neuroradiology between 2011 and 2013, nine individuals were prospectively included between 2014 and 2022 and underwent DTI at the Life & Brain Center. Together, 14 individuals with RE were included in this study. The RE group included nine cases with typical early-onset RE (≤6 years) and five cases with late-onset RE (>6 years) according to Bien *et al.*^27^ The median age at onset was 6 years (range 4–22), and the median disease duration at the date of MRI acquisition was 5 years (range 0–42). Ten of 14 individuals received at least one type of immunotherapy. IVIG was used in most cases (7/14), other substances used were tacrolimus (5/14), glucocorticoids (4/14), azathioprine (1/14), mycophenolate mofetil (1/14) and methotrexate (1/14). Hemispherotomy was considered in all cases, however, it was performed only in 6/14 cases. Further clinical and demographic characteristics are provided in Table 1 and Supplementary Table 1.

### People with RE versus healthy control subjects

Across all people with RE, we found fixels with significantly lower (FWE-corrected *P* < 0.05) FDC values as compared to control subjects corresponding to the corticospinal tract, the cingulate gyrus, the arcuate fascicle, and the superior and inferior longitudinal fascicle in the ipsilesional hemisphere. Concerning interhemispheric fibres, we observed fixels with significantly lower FDC in RE in all parts of the corpus callosum. Changes in FDC were driven by both FD and FC. There were no fixels with significantly higher FDC in people with RE relative to control subjects.

When comparing individuals with RE at different time points after disease onset to control subjects, we observed significantly lower FDC in fixels corresponding to the anterior corpus callosum and the ipsilesional inferior longitudinal fascicle within 2 years after disease onset. In the group between 2 and 6 years after disease onset, fixels with lower FDC could also be observed in the body of the corpus callosum, the corticospinal tract, cingulate gyrus and the arcuate fascicle in the ipsilesional hemisphere.

Using network-based statistics, we found a significantly lower EW in people with RE relative to control subjects for 57 intrahemispheric edges in the ipsilesional, 29 intrahemispheric edges in the contralesional hemisphere and 104 interhemispheric edges. We observed no edges with significantly higher EW in people with RE relative to control subjects.

There were no significant differences in global efficiency between ipsilesional, contralesional and pooled control hemispheres (all pairwise *t*-tests *P* > 0.26).

### Disease duration

**Figure 3:**
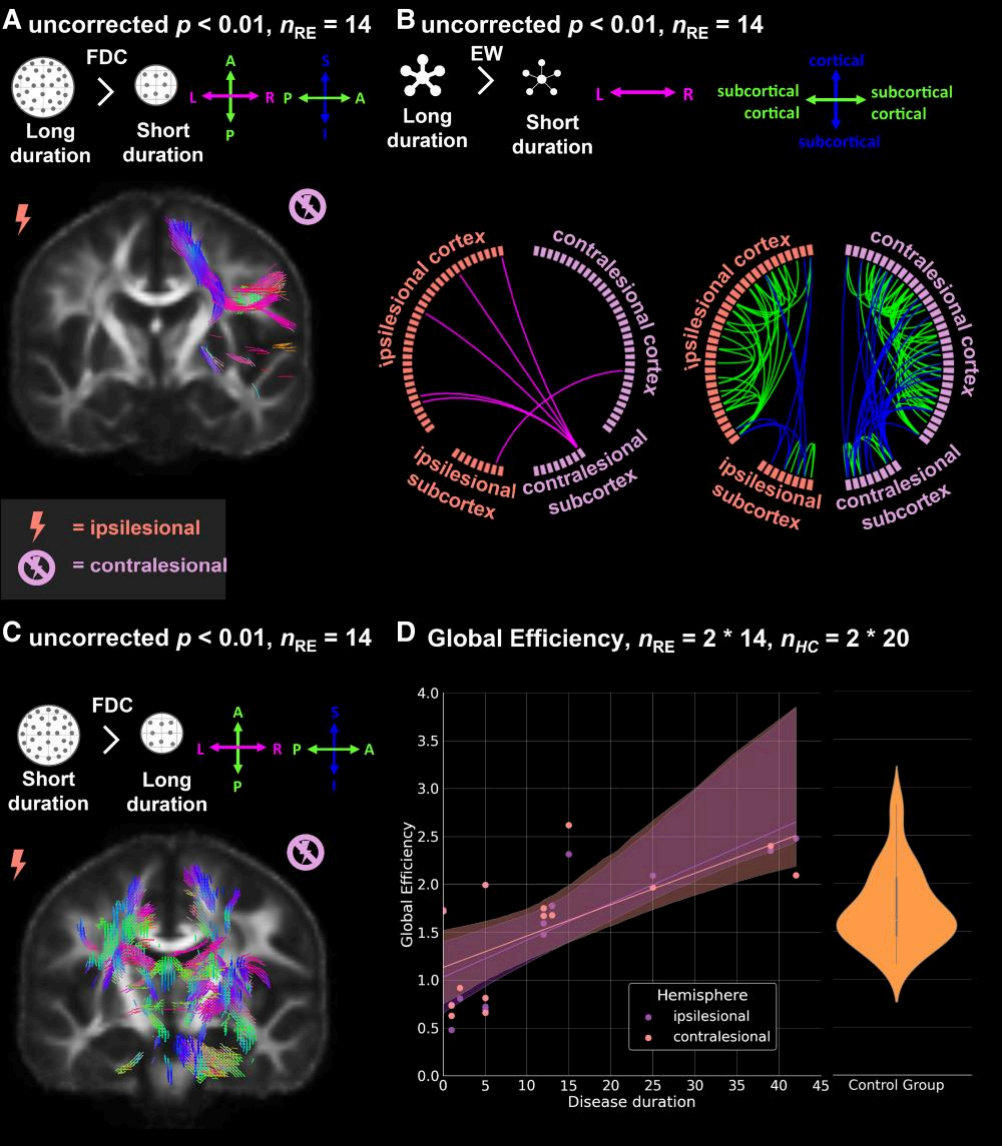
**Effect of disease duration on fibre density and cross-section (FDC), EW and global efficiency.** (**A**) Fixels with significant (uncorrected *P* < 0.01) increase in FDC in individuals with longer disease duration (*n*_RE_ = 14), (**B**) Significant increase (uncorrected *P* < 0.01) in EW in subjects with longer disease duration (*n*_RE_ = 14). (**C**) Fixels with significant (uncorrected *P* < 0.01) decrease in fibre density and cross-section in subjects with longer disease duration (*n*_RE_ = 14), (**D**) Regression of global efficiency by disease duration for ipsi- and contralesional hemispheres. Two hemispheres per individual with Rasmussen’s encephalitis (*n*_RE_ = 2 * 14). Violinplot of global efficiency of pooled control hemispheres. Two hemispheres per healthy control subject (*n*_HC_ = 2 * 20). In (**A/C**), significant fixels are coloured by direction and shown on the population-specific fractional anisotropy template sliced in the coronal plane. FDC, fibre density and cross-section; EW, edge weight; *n*_RE_, number of individuals with Rasmussen’s encephalitis; *n*_HC_, number of healthy control subjects.

Across all people with RE (see Fig. 3), on one hand, we found a significant decrease in FDC (uncorrected *P* < 0.01) with longer disease duration in both the ipsi- and the contralesional hemisphere. These include fixels in the corticospinal tract, the superior longitudinal fascicle, and the cingulum in the ipsilesional hemisphere. In the contralesional hemisphere, this involves fixels in the corticospinal tract, the arcuate fascicle and the superior cerebellar peduncle, as well as crossing fibres in the isthmus and posterior body of the corpus callosum. On the other hand, we observed a significant increase in FDC with longer disease duration of fixels in the superior longitudinal fascicle, corticospinal tract and adjacent association fibres connecting to the inferior frontal gyrus in the contralesional hemisphere. We found no significant increase in FDC in the ipsilesional hemisphere or in interhemispheric fibres.

In network-based statistics, we observed a significant increase in EW in 37 intrahemispheric edges in the ipsilesional hemisphere including seven edges between cortical and subcortical structures. In the contralesional hemisphere, we found 68 intrahemispheric edges, including 23 edges between cortical and subcortical structures, with significantly increased EW. For interhemispheric connections, we found six edges with significantly increased EWs, five of them connecting the contralesional cerebellum to the cortex of the ipsilesional hemisphere. We found no edges featuring significantly decreased EW with longer disease duration.

Regarding network efficiency, we found a positive correlation between disease duration and the global efficiency of both the ipsilesional (*r* = 0.74, *P* = 0.001) and contralesional (*r* = 0.67, *P* = 0.012) hemispheres (see also Fig. 3).

### Age at onset

**Figure 4:**
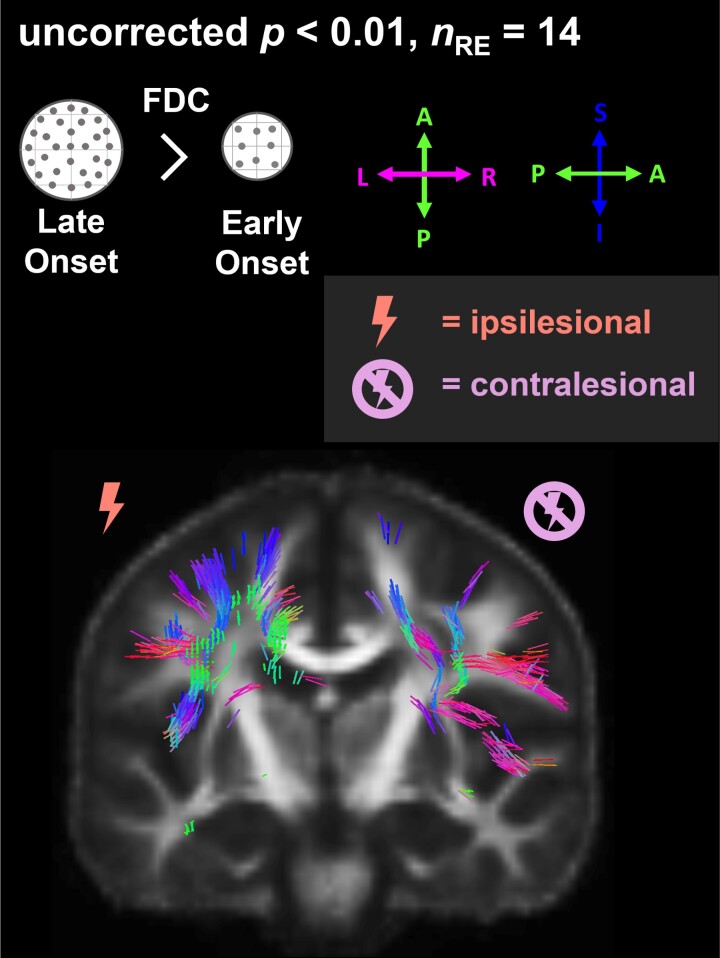
**Effect of age at disease onset on fibre density and cross-section (FDC).** Fixels with significantly (uncorrected *P* < 0.01) lower FDC with younger age at disease onset (*n*_RE_ = 14) are coloured by direction and shown on the population-specific fractional anisotropy template sliced in the coronal plane. FDC, fibre density and cross-section; *n*_RE_, number of individuals with Rasmussen’s encephalitis.

Across all individuals with RE (see also Fig. 4), we found fixels with significantly (uncorrected *P* < 0.01) lower FDC with a younger age at onset located in the corticospinal tract, inferior and superior longitudinal fascicle, arcuate fascicle and the cingulum. In the contralesional hemisphere, fixels with significantly lower FDC with an earlier age at onset correspond to association fibres of the motor cortex, the superior and inferior longitudinal fascicle, and involve crossing fibres of the corpus callosum midbody, isthmus and splenium. While there is a higher number of fixels in the postcentral area in the ipsilesional hemisphere, the majority of corticospinal fixels in the contralesional hemisphere connect to the precentral area. Few fixels showing significantly higher FDC with younger age at onset were spuriously distributed in the white matter of the contralesional hemisphere.

In network-based statistics, we found three edges with significantly lower and one edge with significantly higher EW with younger age at disease onset.

## Discussion

The aim of this study was to investigate white matter microstructural and network-level connectivity alterations in individuals with RE. Our results revealed both reduced white matter microstructural integrity and network-level connectivity primarily in ipsilesional and crossing fibres. While interhemispheric fibres are already affected at the very beginning of the disease, linear regression with disease duration showed microstructural degradation in the ipsilesional hemisphere, and both microstructural degradation and tracts with increased microstructural integrity contralaterally. On network level, reorganisation with increased intrahemispheric connectivity and global efficiency with disease duration was observed in both hemispheres.

### Interhemispheric tracts are affected prior to ipsilesional intrahemispheric connections

While the extensive intrahemispheric microstructural and network-level alterations in the ipsilesional hemisphere in people with RE are not surprising, the prominent involvement of interhemispheric connections is remarkable. Of note, these connections are the first to be microstructurally damaged in the acute phase, even earlier than major ipsilesional white matter association tracts, such as the superior longitudinal fascicle or the cingulum. As the disease progresses, this microstructural damage spreads into the frontal (genu and premotor) and eventually to the body and splenium parts of the corpus callosum, affecting the entire structure. This aligns with the observation that cortical atrophy often occurs first in insular and frontotemporal regions, from where interhemispheric tracts pass through these parts of the corpus callosum.^28-30^

Beyond this, a closer look at the interhemispheric connections is particularly relevant, as recent studies suggest involvement of the contralesional hemisphere. Morphometric MRI analyses found both lower and higher cortical volumes in the contralesional hemisphere, also, epileptiform EEG abnormalities over the contralesional hemisphere have repeatedly been reported. These observations, however, could so far not be aetiologically explained. There are essentially two explanations for secondary degeneration of the contralesional hemisphere: Wallerian degeneration or transhemispheric diaschisis.^2,8,28,30,31^ Wallerian degeneration is a form of axonal degeneration that can occur in both the central and peripheral nervous systems. It is characterised by axonal degradation in parts of the axon distal to the initial injury.^32^ An initial insult, which in the case of RE would be mainly inflammation in the ipsilesional hemisphere, would cause the entire axon of the affected neurons to degenerate across the midline as well. The microstructural degeneration of interhemispheric fibres, as it is evident in our results, could be an imaging correlate of axonal damage in the sense of Wallerian degeneration. In contrast, the concept of transhemispheric diaschisis has first been derived from post-stroke research. It describes the phenomenon that regions of the brain that are functionally connected but anatomically distant from the primary lesion degenerate as a result of deafferentation.^10^ Since our results show a significant reduction of interhemispheric connectivity, this makes it seem likely that cortical degeneration in the contralesional hemisphere as seen in previous studies is caused by transhemispheric diaschisis.^3^

### Bilateral microstructural degeneration, but intrahemispheric reorganisation with disease duration

During the course of the disease, further microstructural degeneration occurs not only in the ipsilesional, but almost symmetrically in the contralesional hemisphere. Previous studies have found epileptiform EEG abnormalities not only over the ipsilesional, but also over the contralesional hemisphere.^4-7^ Cognitive deterioration attributable to the contralesional hemisphere has been interpreted as a functional correlate of contralesional epileptiform activity,^5^ and our data suggest that progressive microstructural degeneration in both hemispheres is a structural correlate of epileptic activity. This aligns with previous studies that interpreted lower FDC in temporal lobe epilepsy as a consequence of seizures.^33^

However, during the course of the disease and besides bilateral microstructural degeneration, FDC in the contralesional corticospinal tract increases. In addition to this, increased network-level EWs and increased global efficiency show that intrahemispheric network remodelling occurs in both hemispheres. While this was expected in the contralesional hemisphere, where an increase in cortical volume has also been reported and interpreted as compensatory neuroplasticity,^2^ the increase in EW and global efficiency in the ipsilesional hemisphere may seem counterintuitive at first. We speculate that the increase in intrahemispheric integration on network level is due to the reduced microstructural integrity in the corpus callosum. With microstructural degradation of interhemispheric fibres, these are impaired in their ability to relay information efficiently and rapidly. As a consequence, both hemispheres would become functionally isolated, which would ultimately trigger intrahemispheric network-level remodelling in both hemispheres to compensate for the loss of input from the other hemisphere. As a note of caution, it should be mentioned that effects due to individual therapeutic approaches (both immunotherapy and anti-seizure medication) are not considered by the statistical model. Although azathioprine does not seem to affect brain morphology,^34^ it cannot be ruled out that other immunomodulatory treatments may have effects on white matter integrity. A confounding effect of anti-seizure medication must also be assumed.^35^

### Age at onset plays a role in white matter alterations

Previous research has shown that the clinical features of typical childhood-onset RE and late-onset RE are very different. On microstructural level, early onset is associated with inferior microstructural integrity in both hemispheres, predominantly in intrahemispheric fibres such as the corticospinal tract or superior longitudinal fascicle. These findings reflect the clinical characteristics of the two RE types: Late-onset RE is often described as being milder, with a slower disease progression and less severe neurological and cognitive deficits.^27,36-38^ Also, cortical atrophy rates in the ipsilesional hemisphere are reportedly lower in late-onset RE and contralateral MRI abnormalities are only subtle at best.^37^ In contrast, the probability for involvement of the entire hemisphere increases with an earlier age at onset and childhood RE presents with more severe neurological and cognitive deficits.^39^ Contralesional epileptiform activity is also more likely in childhood RE, which aligns well with our finding of bilateral microstructural involvement specifically in people with RE and early disease onset. Furthermore, this observation may explain why the rare cases of bilateral RE almost exclusively occur with childhood-onset.^40,41^

### Strengths and limitations

This study has two major limitations. The first limitation is the design of the study: A retrospective study is always challenged by the complexity of possible confounding effects of therapeutic interventions, such as anti-seizure medication and immunomodulatory treatments. The second limitation is the small sample size. Although this study showed significant differences even within the RE group, it is safe to say that a larger sample size, although difficult to obtain due to the low prevalence of RE, would provide more insights into the underlying characteristics and effects of RE on white matter. Nevertheless, to the best of our knowledge, this is the first study to investigate white matter changes in RE patients and to provide further insight into white matter properties along the disease course of RE. Our 3-fold approach provides a robust, multiscale insight into white matter connectivity from a microstructural, local and global, and network-level perspective.

## Conclusion

By analysing microstructural and network properties of white matter tracts in RE, our study shows that interhemispheric connections are affected early in the disease course, which could explain the suspected involvement of the ‘unaffected’ contralesional hemisphere. While microstructural damage progresses bilaterally during the disease course, increased intrahemispheric connectivity and global efficiency are seen on network level, which may fall under the realm of compensatory neuroplasticity. Altogether, our results show that RE is in two regards a network-wide disorder that is not limited to the cortex of the ipsilesional hemisphere: On the one hand, white matter connections across the entire brain are affected by the pathological degenerative processes, and on the other hand, alterations on network level show signs of compensatory remodelling.

## Supplementary Material

fcad290_Supplementary_Data

## Data Availability

The data that support the findings of this study are available on request from the corresponding author. The data are not publicly available due to privacy or ethical restrictions.
