# Supplementary material for: Globally altered microstructural properties and network topology in Rasmussen’s encephalitis
Source: Brain Commun. 2023 Nov 1;5(6):fcad290. doi: 10.1093/braincomms/fcad290 (PMC10638105; doi:10.1093/braincomms/fcad290)
Supplement: fcad290_Supplementary_Data [file fcad290_supplementary_data.pdf]

Supplementary Material

No.	Sex	Ipsilesional Hemisphere	Age at Onset	Age at Scan	Disease duration at Scan
1	m	right	4	16y	12y 2m
2	f	left	6y	49y	42y 10m
3	f	left	6y	19y	13y 1m
4	f	right	13y	25y	12y 6m
5	f	left	22y	37y	15y 0m
6	f	right	6y	46y	39y 8m
7	f	left	8y	8y	0y 8m
8	f	left	5y	7y	1y 6m
9	f	right	5y	7y	1y 8m
10	m	left	5y	10y	5y 5m
11	f	right	22y	24y	2y 1m
12	f	right	6y	12y	5y 4m
13	f	left	8y	14y	5y 3m
14	m	left	21y	47y	25y 10m

Supplementary Table 1. Demographic data. Abbreviations: f: female, m: male, y: years, m: months.

Image type	n scans	model	No. of Gradient Directions	B-Values	TR (s)	TE (s)	flip angle	voxel size
T1	**20**	**Siemens Magnetom Trio**			**1.66**	**0.00254**	**9**	**0.8 x 0.8 x 0.8**
	**4**	**Siemens Magnetom Trio**			**1.57**	**0.00342**	**15**	**1 x 1 x 1**
	**4**	**Philips Healthcare Ingenia**			**0.008**	**0.003**	**8**	**1 x 1 x 1**
DTI	**20**	**Siemens Magnetom Trio**	**66**	**1000**	**9**	**0.087**	**90**	**1.71 x 1.71 x 1.7**
	**3**	**Siemens Magnetom Trio**	**66**	**1000**	**12**	**0.1**	**90**	**1.71 x 1.71 x 1.7**
	**1**	**Philips Healthcare Ingenia**	**33**	**1000**	**7**	**0.06**	**90**	**0.8 x 0.8 x 2**
	**1**	**Philips Healthcare Ingenia**	**33**	**1000**	**12**	**0.06**	**90**	**1 x 1 x 2**
	**1**	**Philips Healthcare Ingenia**	**33**	**1000**	**13**	**0.06**	**90**	**1 x 1 x 2**
	**1**	**Philips Healthcare Ingenia**	**33**	**1000**	**13**	**0.08**	**90**	**1 x 1 x 2**
	**1**	**Philips Healthcare Ingenia**	**33**	**800**	**6.9**	**0.06**	**90**	**1 x 1 x 2**

Supplementary Table 2. Structural and Diffusion Tensor Imaging (DTI) acquisition parameters. Abbreviations: DTI: Diffusion Tensor Imaging, TR: Repetition Time, TE: Echo Time.

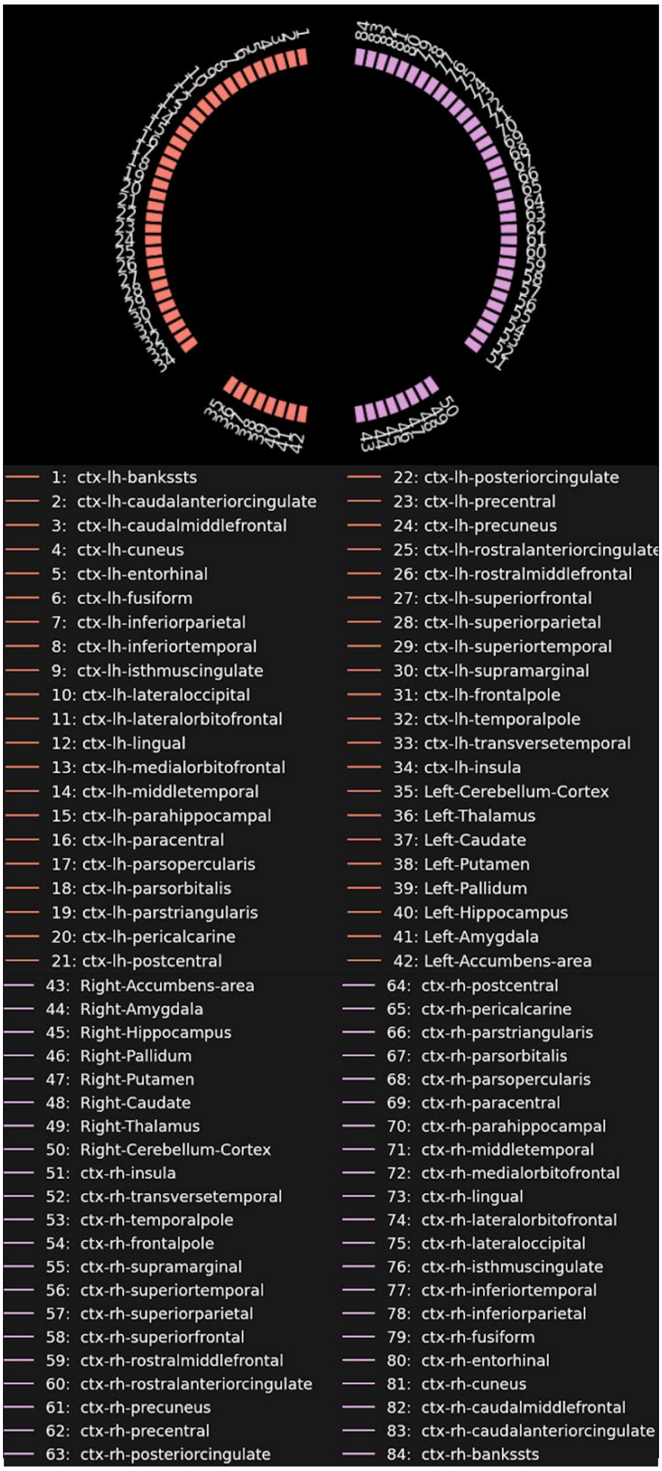

Supplementary Figure 1. Anatomical label descriptions for structural connectivity circles based on the Desikan-Killiany Atlas. Further information [doi:10.1016/j.neuroimage.2006.01.021 (harvard.edu)]
